# UK recommendations for *SDHA* germline genetic testing and surveillance in clinical practice

**DOI:** 10.1136/jmedgenet-2021-108355

**Published:** 2022-03-08

**Authors:** Helen Hanson, Miranda Durkie, Fiona Lalloo, Louise Izatt, Terri P McVeigh, Jackie A Cook, Carole Brewer, James Drummond, Samantha Butler, Treena Cranston, Ruth Casey, Tricia Tan, Daniel Morganstein, Diana M Eccles, Marc Tischkowitz, Clare Turnbull, Emma Roisin Woodward, Eamonn R Maher, UK Cancer Genetics Centres, Alan Donaldson

**Affiliations:** 1 South West Thames Regional Genetic Services, St George’s University Hospitals NHS Foundation Trust, London, UK; 2 Division of Genetics and Epidemiology, Institute of Cancer Research, London, UK; 3 Sheffield Diagnostic Genetics Service, Sheffield Children’s NHS Foundation Trust, North East and Yorkshire Genomic Laboratory Hub, Sheffield, UK; 4 Clinical Genetics Service, Manchester Centre for Genomic Medicine, Central Manchester University Hospitals NHS Foundation Trust, Manchester, UK; 5 Clinical Genetics, Guy's and St Thomas' NHS Foundation Trust, London, UK; 6 Cancer Genetics Unit, Royal Marsden NHS Foundation Trust, London, UK; 7 Department of Clinical Genetics, Sheffield Children's NHS FoundationTrust, Sheffield, UK; 8 Department of Clinical Genetics, Royal Devon and Exeter NHS Foundation Trust, Exeter, UK; 9 East NHS Genomic Laboratory Hub, Cambridge University Hospitals Genomic Laboratory, Cambridge University Hospital Foundation Trust, Cambridge, UK; 10 Molecular Genetics, West Midlands Regional Genetics Laboratory, Birmingham, West Midlands, UK; 11 Oxford Molecular Genetics Laboratory, Churchill Hospital, Oxford, UK; 12 Department of Endocrinology, Cambridge University Hospitals NHS Foundation Trust, Cambridge, UK; 13 Department of Medical Genetics, University of Cambridge and Cambridge University Hospitals NHS Foundation Trust, Cambridge, UK; 14 Section of Investigative Medicine, Imperial College London, London, UK; 15 Endocrinology, Royal Marsden Hospital NHS Trust, London, UK; 16 Cancer Sciences Unit, Faculty of Medicine, University of Southampton, Southampton, UK; 17 Division of Evolution and Genomic Sciences, School of Biological Sciences, Faculty of Biology Medicine and Health, The University of Manchester, Manchester, UK

**Keywords:** endocrinology, genetic counselling, genetic predisposition to disease, genetic testing

## Abstract

*SDHA* pathogenic germline variants (PGVs) are identified in up to 10% of patients with paraganglioma and phaeochromocytoma and up to 30% with wild-type gastrointestinal stromal tumours. Most *SDHA* PGV carriers present with an apparently sporadic tumour, but often the pathogenic variant has been inherited from parent who has the variant, but has not developed any clinical features. Studies of *SDHA* PGV carriers suggest that lifetime penetrance for SDHA-associated tumours is low, particularly when identified outside the context of a family history. Current recommended surveillance for *SDHA* PGV carriers follows an intensive protocol. With increasing implementation of tumour and germline large panel and whole-genome sequencing, it is likely more *SDHA* PGV carriers will be identified in patients with tumours not strongly associated with *SDHA,* or outside the context of a strong family history. This creates a complex situation about what to recommend in clinical practice considering low penetrance for tumour development, surveillance burden and patient anxiety. An expert *SDHA* working group was formed to discuss and consider this situation. This paper outlines the recommendations from this working group for testing and management of *SDHA* PGV carriers in clinical practice.

## Introduction

*SDHA* likely pathogenic or pathogenic germline variants (ie, class 4 or class 5 variants according to ACMG/AMP criteria and henceforth collectively referred to as PGVs) are identified in up to 10% of patients with phaeochromocytoma and paraganglioma (PPGL) and can account for up to 50% of SDH-deficient wild-type gastrointestinal stromal tumours (wtGISTs) or up to 30% of wtGIST.^1^ wtGIST are a unique and uncommon subtype of GISTs that lack somatic-activating variants in the tyrosine kinase c-KIT or platelet-derived growth factor receptor alpha receptors. Most individuals with a PGV in *SDHA* (henceforth referred to as *SDHA* PGV carriers) present with an apparently sporadic tumour without relevant family history, and most PGVs are inherited from a parent who has not presented with any clinical features.^2^

A large study of patients with paraganglioma (PGL) from the Netherlands, including 30 index *SDHA* PGV carriers and 56 non-index *SDHA* PGV carriers, highlighted that the tumour penetrance is low in non-index *SDHA* PGV carriers, with penetrance at age 70 years estimated to be 10%.^3^ In addition, though penetrance in *SDHA* PGV carriers was estimated as 39% at 40 years of age in a prospective study of a population-based registry of patients with PPGL, there was a significant difference in index patients (45%, n=29) versus *SDHA* carrier relatives (13%, n=9; p<0.001).^4^ Lower estimates of penetrance (~1.7%) have been reported using a Bayesian approach looking at *SDHA* PGVs in EXAC data and patients with PGL and in a cohort of *SDHA* PGV carriers (~0.1%–4.9%).^5 6^ *SDHA* NM_004168.4: c.91C>T p.(Arg31*) accounts for a large proportion of known carriers and also occurs at frequency of 0.04% (53/128 900) in non-Finnish Europeans (https://gnomad.broadinstitute.org/variant/5-223624-C-T, accessed 15 November 2021).^3^ Despite this frequency there is currently no evidence to suggest that *SDHA* c.91C>T p.(Arg31*) has a different penetrance to any other *SDHA* PGVs and variant specific management is not advocated by other groups.^7^

While published literature suggests that most *SDHA* PGV carriers will not manifest *SDHA*-related tumours, thus calling into question the clinical utility of surveillance in this low-risk group, probands with *SDHA*-related tumours and confirmed *SDHA* PGVs typically present at young ages (median age at diagnosis is 28 years with a range of 8–76 years^4^). In addition to malignant wtGIST, *SDHA* PGV carriers may also develop malignant PGL.^8^ As for many other rare cancer predisposition genes, there is sparse information available on the effectiveness of surveillance in *SDHA* PGV carriers and the impact of early detection on clinical outcomes.^9^

At the UKCGG (UK Cancer Genetics Group) Consensus meeting in Cambridge in Spring 2019 (https://www.ukcgg.org/information-education/ukcgg-consensus-meetings/), a surveillance protocol for *SDHA* PGV carriers was agreed consisting of annual clinical review and biochemistry with abdominal imaging and MRI neck, thorax, abdomen and pelvis at baseline, followed by 3–5 yearly imaging, based on published recommendations and expert opinion.^10 11^

Since that meeting, questions regarding the utility of predictive testing and surveillance for a low penetrance condition have been raised in national forums and highlighted by *SDHA* PGVs being identified as secondary findings through the 100,000 genomes project or through wider panel testing in probands with phenotypes not directly related to *SDHA.* 12 It was therefore agreed timely to revisit predictive testing and surveillance guidelines specifically for *SDHA* to address these complex clinical issues.

## Methods

A preliminary scoping survey was sent out to the 24 UK Regional Genetics centres in July 2020, to establish current practice. There was a total of 24 individual responses from 18 centres. The results of the survey were collated and discussed further by the *SDHA* working group in a virtual meeting and draft recommendations proposed following this meeting. These recommendations were then circulated to three endocrinologists and subsequently to a representative from each of the 24 Regional Genetics centres. Further input was sought from UK laboratories undertaking *SDHA* testing, specifically regarding reporting of *SDHA* PGVs and the final recommendations agreed by the working group.

## Recommendations

For these recommendations, it was agreed to define clear ‘on-target’ *SDHA-*associated tumours based on published literature and expert group opinion to make practical recommendations regarding reporting, clinical management and predictive testing (the terms on-target and SDHA-associated tumours are synonymous, but the term SDHA associated has been used through this document).^4 13–15^ Tumours not specifically listed in this table are currently considered to be ‘off-target’ tumours (table 1). While it is recognised that *SDHA* PGVs may potentially contribute to a wider phenotypic tumour spectrum, current evidence for further clear associations beyond the tumours defined in table 1 is limited.^13^

**Table 1:** Succinate-deficient tumours associated with *SDHA* PGV (SDHA-associated/on-target tumours)

Tumour type	Strength of association
Wild-type GIST	+++
Paraganglioma*	++
Phaeochromocytoma*	++
Renal cancer†	+
Neuroblastoma‡	Rare
Pituitary adenoma‡	Rare

*For PPGL it is assumed that PGV in other PPGL predisposition genes have been excluded. The National Test Directory now indicates that this testing should include *FH*, *MAX*, *MEN1*, *RET*, *SDHA*, *SDHAF2*, *SDHB*, *SDHC*, *SDHD*, *TMEM127* and *VHL* including analysis for CNVs (National Test Directory indication R223 Inherited phaeochromocytoma and PGL.^17^

†For renal cancer, histopathological examination for characteristic features and immunohistochemistry can be helpful in assessing dSDH status.^26^ SDHB expression is lost in most dSDH tumours with a germline *SDHA*, *SDHB*, *SDHC* or *SDHD* pathogenic variant. Limitation to the utility of SDHB IHC include interobserver variation, false-negative results (presence of SDHB on IHC where a germline variant exists may be more common for *SDHA*-mutated tumours) and equivocal SDHB staining patterns in the presence of germline or somatic VHL inactivation.^11^ SDHA IHC can reveal loss of SDHA expression in SDHA-mutated tumours but is less widely available than SDHB IHC.

‡For neuroblastoma and pituitary adenoma, immunohistochemical evidence of SDH deficiency should be sought and other causes excluded before an *SDHA* variant is considered causal.

GIST, gastrointestinal stromal tumour; IHC, immunohistochemistry; PGL, paraganglioma; PGV, pathogenic germline variant; PPGL, phaeochromocytoma and paraganglioma.

### Recommendation 1: identification of an *SDHA* PGV in an individual with an *SDHA*-associated tumour

When diagnostic genetic testing is undertaken in an individual with wtGIST, PPGL, renal cancer, neuroblastoma or pituitary tumour and a PGV identified, including *SDHA* c.91C>T p.(Arg31*), provided other causes have been excluded or there is appropriate immunohistochemical evidence (see table 1 footnotes), the PGV can be considered to be associated with the clinical phenotype and the diagnostic laboratory report should reflect this. However, we would recommend that the report highlights the low penetrance of *SDHA* PGVs and the need for onward referral to a clinical genetics service for the discussion of predictive genetic testing (table 2). Suggested wording approved by the UK Can-VIG group^16^ is ‘*SDHA* pathogenic germline variants appear to have a very low penetrance in asymptomatic relatives who are heterozygous for the variant. Therefore, while predictive testing in other family members may be offered, we would recommend referral to clinical genetics for further discussion’.

**Table 2:** Summary of recommendations

	Report germline likely pathogenic/pathogenic variant in diagnostic setting	Surveillance* for affected proband	Offer predictive testing	Offer surveillance* if positive predictive test
Individual with *SDHA-*associated tumour (wtGIST, paraganglioma, phaeochromocytoma), renal cancer, neuroblastoma and pituitary adenoma with immunohistochemical evidence of SDH deficiency, see table 1)	Yes	Yes Offer follow-up for initial tumour and surveillance for metachronous tumours (see recommendation 4)	Recommend for FDR, following detailed discussion	Offer surveillance (see recommendation 6) following detailed discussion regarding current knowledge and limitations
Individual with non-*SDHA*-associated tumour	Yes, recommend reporting is coupled with a recommendation that SDH IHC is performed and the finding is considered to be a non-actionable secondary finding unless there is immunohistochemical evidence of SDHB/SDHA loss in the tumour or a family history of SDHA-associated tumours†	No†	No†	N/A

*Surveillance: annual symptom review, blood pressure monitoring, biochemistry with plasma metanephrines and 3–5 yearly imaging of neck, thorax, abdomen and pelvis, preferably with MRI from age 15.

†For individuals with non-SDHA-associated tumours identified to have a PGV in *SDHA*, we would consider these a non-clinically actionable finding, unless the tumour is demonstrated to show SDHB/SDHA loss or there is a family history of SDHA-associated tumours. If either IHC loss or family history is confirmed, then recommendations should shift to that for an individual with an SDHA-associated tumour.

FDR, first-degree relative; IHC, immunohistochemical; PGV, pathogenic germline variant; wtGIST, wild-type gastrointestinal stromal tumour.

### Recommendation 2: identification of an *SDHA* PGV in an individual with a non-*SDHA*-associated tumour

In most situations within current UK clinical practice, germline *SDHA* testing will only be requested for an individual with a personal or family history of *SDHA*-associated tumours (currently indications R223 and R363 in the current National Genomic Test Directory, October 2021).^17^

However, with increasing use of large germline cancer predisposition gene panels, large somatic solid tumour panels and paired whole-genome sequencing (WGS), *SDHA* PGVs may also be identified in off-tumour settings, that is, in individuals with tumours not listed in table 1.

When an *SDHA* PGV is identified during tumour analysis, the likelihood of it being of germline origin is high in both on-tumour (associated tumour types) and off-tumour (non-associated tumour types) settings. Where an *SDHA* pathogenic variant has been identified in tumour tissue, germline testing has been recommended by the European Society of Medical Oncology Precision Medicine Working Group.^18^ However, identification of an *SDHA* PGV in an individual with a non-*SDHA*-associated tumour could be considered a secondary or incidental finding. ACMG Recommendations for Reporting of Incidental Findings in Clinical Exome and Genome Sequencing, which are supported and followed within UK practice, suggest that these findings are not reported.^19^

We would suggest that if an *SDHA* PGV is identified in an individual with cancer but in an off-tumour setting, for example, through WGS or extended gene panel testing, that the PGV is reported but coupled with a recommendation that SDH immunohistochemistry (IHC) is performed and the finding considered to be a non-actionable secondary finding unless there is immunohistochemical evidence of SDHB/SDHA protein loss or a family history of SDHA-associated tumours (table 2).

The working group felt that due to the low penetrance of *SDHA* PGVs outside the context of a personal or family history of SDHA-associated tumours, in this situation we would not recommend any surveillance in affected individuals or predictive testing for other family members (table 2). Should there be immunohistochemical evidence of SDHB/SDHA loss, or suggestive family history, then we would recommend surveillance and predictive testing be undertaken in line with the recommendations for patients with *SDHA*-associated tumours (see recommendations 4–6 below).

### Recommendation 3: Identification of an *SDHA* PGV in an individual without cancer

In some situations where an individual has had germline genetic testing for another indication but does not have a personal history of cancer, an *SDHA* PGV may be identified. ACMG Recommendations for Reporting of Incidental Findings in Clinical Exome and Genome Sequencing, which are supported and followed within UK practice, suggest that these findings are not reported.^19^

### Recommendation 4: surveillance for *SDHA* PGV carriers affected with *SDHA*-associated tumours

For an *SDHA* PGV carrier with an SDHA-associated tumour, with respect to the primary tumour, we would recommend at the very least annual clinical examination to include blood pressure assessment and biochemistry to include plasma metanephrines, combined with imaging of the original tumour region (eg, abdomen if phaeochromocytoma, abdominal PGL, GIST or renal tumour) if complete resection was achieved. Ongoing follow-up, surveillance and discharge for their original diagnosis should be determined by multidisciplinary team decision depending on the clinical details and consideration of relevant published guidelines for the follow-up of PPGL.^20^

There are minimal data on the occurrence of a second tumour in individuals with an *SDHA* PGV. However, metachronous tumours have been reported, in single case reports and in 4 out of 21 index cases in a study from the Netherlands.^3 8^

With regards to ongoing surveillance for metachronous tumours, based on UKCGG Consensus guidelines,^21^ we would recommend annual symptom review, blood pressure monitoring and biochemistry with plasma metanephrines (with 24-hour urinary metanephrines an alternative especially in children). Imaging should include neck, thorax, abdomen and pelvis at baseline, followed by 3–5 yearly surveillance, preferably with MRI, recognising that follow-up of the original tumour region may be more frequent. Ongoing follow-up should take place in an Endocrinology clinic or Joint Endocrine-Genetics clinic.

The UKCGG guidelines did not make recommendations for pituitary surveillance. This is in part due to the low penetrance for pituitary adenoma, but also the relatively high rate of incidental detection of non-functional microadenoma, estimated to be 10% or greater in a healthy population.^22^ At present, we would not recommend routine pituitary imaging, in line with a recent international consensus guideline on surveillance in SDHx PGV carriers.^7^ Consideration of annual prolactin and IGF1 and requesting that the MRI neck includes a cut through the pituitary has been proposed as a surveillance recommendation.^11^ At present, we would only recommend this within the context of a trial or service evaluation and suggest that recommendations are revisited in the future as more data become available.

### Recommendation 5: predictive *SDHA* testing

A limited number of small studies have demonstrated that the penetrance of *SDHA* in non-probands is likely to be low: 10%–13% lifetime risk in two studies.^3 4^ These data have raised the question of the utility of predictive testing and surveillance for this patient group. Concern has been raised about the potential to increase anxiety for these families when lifetime risk of an *SDHA*-associated tumour may be low. However, patients can present with aggressive and metastatic disease which may be avoided by early detection. The lifetime penetrance in non-proband family members is also higher than in penetrance estimates from comparing *SDHA* allele frequencies in affected individuals and population controls, ~2%.^5 6^

The survey of UK Geneticists and Endocrinologists suggested that at present while there is recognition that the cancer risk for non-proband family members may be low, it was felt that the data in this area are limited and there was concern about not discussing predictive testing with family members. There was agreement that while there are limited data available, predictive testing and surveillance should be discussed within the context of our current knowledge, but that this should be reviewed as and when new data on penetrance and effectiveness of surveillance are available.

We would recommend that predictive genetic testing should be considered in families where an *SDHA* PGV has been identified in an individual with an *SDHA*-associated tumour (see tables 1 and 2) or an individual with an off-target tumour not typically associated with SDHA, but with suggestive IHC loss or family history of SDHA-associated tumours (table 2). Due to the reported low penetrance and few reports of familial cases we suggest consideration of offering predictive testing only to first-degree relatives of an affected proband, unless there is a wider family history of SDHA-associated tumours (table 2).

We suggest that detailed discussion regarding predictive testing with these families should take place, ensuring patients are actively involved in the decision-making process regarding predictive testing and a joint decision made whether to proceed. We would recommend particular attention and clear discussion of the low penetrance of *SDHA* PGVs, counselling of the lack of clear evidence on utility of surveillance and the potential for incidental findings.

We would also recommend detailed discussion of symptoms relating to SDHA-associated tumours, both as an adjunct or alternative to surveillance. We suggest not actively offering predictive testing to second-degree relatives; however, we would recommend providing information on symptoms relating to SDHA-associated tumours and the importance of seeking a specialist opinion if they have specific symptoms, for example, hypertension, severe recurrent headaches, dyspepsia or upper gastrointestinal bleeding or unintentional weight loss.

### Recommendation 6: surveillance for unaffected *SDHA* PGV carriers

As for many other cancer predisposition syndromes, comprehensive data regarding the clinical utility of surveillance are limited in *SDHA* PGV carriers. However, it is recognised that patients can present at a young age with metastatic disease and cases of positive surveillance have been reported.^9 23^ There is controversy both over whether surveillance should be offered and if it is, the extent of the surveillance. Surveillance with plasma metanephrines alone may not detect the non-secretory head and neck PGL and therefore, if surveillance is recommended it should comprise both biochemical studies and imaging including neck, thorax, abdomen and pelvis.

There are no studies assessing the optimal surveillance interval, but an early study found tumour doubling time of 4.2 years for head and neck PGLs^24^ and 5.8 years in a series of *SDHB* and *SDHD* patients with head and neck PGLs, whereas abdominal and thoracic PGLs grew more slowly, doubling at 6.94 and 11.8 years, respectively.^25^

Based on expert opinion, UKCGG guidelines in 2019 recommended:

- Annual symptom review, clinical examination to include blood pressure and annual biochemical surveillance with plasma metanephrines (with 24-hour urinary metanephrines an alternative, especially in children) from age 10.
- Radiological surveillance every 3–5 years of neck, thorax, abdomen and pelvis, preferably with MRI from age 15. Where possible, imaging is best performed in centres with experience of surveillance for PPGL. Ultrasound is not recommended.
- Routine pituitary imaging is not recommended (see also Recommendation 4: surveillance for *SDHA* PGV carriers affected with *SDHA*-associated tumours section).

These recommendations are in line with recently published international consensus guidelines for SDHx PGV carriers, who also advise that by the age of 70 years, if individuals are well with no tumours, the interval of imaging can be increased to 5 yearly until age 80 and then stopped if well.^7^ We would suggest that predictive genetic testing is considered from the age that surveillance commences, that is, around 10 years.

## Conclusion and future work

It was recognised that there is currently limited knowledge regarding the full phenotype and penetrance of tumours in individuals with *SDHA* PGVs. Therefore, there is a critical need for systematic prospective data collection to address this and the outcomes of surveillance.

It was also recognised that there has been inconsistency in practice across the country, and it is hoped that these recommendations will help to address this. To improve patient understanding, development of a national patient leaflet is planned to both highlight the uncertainty regarding true cancer risk for this patient group, the pros and cons of predictive testing and surveillance and education on symptoms.

Given the uncertainties and lack of data highlighted in this report, the working group recommend that:

1. Prospective research studies and/or service evaluations are undertaken to define of the natural history of individuals with *SDHA* PGVs identified with SDHA-associated and SDHA non-associated tumours and consider if specific genetic or environmental factors alter the penetrance in *SDHA* carriers.
2. The outcomes and clinical utility of surveillance in *SDHA* PGV carrier probands and non-probands are carefully documented and evaluated.
3. Novel means of early detection are sought to reduce burden on radiology departments.
4. The opinions and preferences of patient groups are canvassed.
